# Prevalence and Predictors of Tuberculosis in Adults and Adolescents With Sputum Trace Ultra Results in 2 High-Burden Clinical Settings

**DOI:** 10.1093/cid/ciag019

**Published:** 2026-03-20

**Authors:** Caitlin Visek, Ronit R Dalmat, Annet Nalutaaya, Kamoga Caleb Erisa, Patrick Biché, Gabrielle Stein, Amanda Ganguloo, Robin Draper, Mariam Nantale, Adrienne E Shapiro, Douglas Wilson, Achilles Katamba, Paul K Drain, Emily A Kendall

**Affiliations:** Division of Infectious Diseases, Department of Medicine, Johns Hopkins University School of Medicine, Baltimore, Maryland, USA; Department of Global Health, University of Washington, Seattle, Washington, USA; Uganda Tuberculosis Implementation Research Consortium, Walimu, Kampala, Uganda; Uganda Tuberculosis Implementation Research Consortium, Walimu, Kampala, Uganda; Department of Epidemiology, Johns Hopkins Bloomberg School of Public Health, Baltimore, Maryland, USA; Department of Global Health, University of Washington, Seattle, Washington, USA; Umkhuseli Innovation and Research Management, Pietermaritzburg, South Africa; Department of Internal Medicine, Harry Gwala Regional Hospital, University of KwaZulu-Natal, Pietermaritzburg, South Africa; Umkhuseli Innovation and Research Management, Pietermaritzburg, South Africa; Department of Internal Medicine, Harry Gwala Regional Hospital, University of KwaZulu-Natal, Pietermaritzburg, South Africa; Uganda Tuberculosis Implementation Research Consortium, Walimu, Kampala, Uganda; Department of Global Health, University of Washington, Seattle, Washington, USA; Umkhuseli Innovation and Research Management, Pietermaritzburg, South Africa; Department of Internal Medicine, Harry Gwala Regional Hospital, University of KwaZulu-Natal, Pietermaritzburg, South Africa; Uganda Tuberculosis Implementation Research Consortium, Walimu, Kampala, Uganda; Clinical Epidemiology & Biostatistics Unit, Department of Medicine, Makerere University College of Health Sciences, Kampala, Uganda; Department of Global Health, University of Washington, Seattle, Washington, USA; Department of Epidemiology, University of Washington, Seattle, Washington, USA; Division of Infectious Diseases, Department of Medicine, Johns Hopkins University School of Medicine, Baltimore, Maryland, USA; Department of Epidemiology, Johns Hopkins Bloomberg School of Public Health, Baltimore, Maryland, USA

**Keywords:** tuberculosis, molecular diagnostic testing, Xpert MTB/RIF Ultra, trace positive

## Abstract

**Background:**

Some patients who test trace positive on Xpert MTB/RIF Ultra (“Ultra”), a highly sensitive molecular diagnostic platform, may not have tuberculosis (TB) disease. A better understanding of the prevalence of TB, associated clinical characteristics, and utility of additional diagnostic tests among people with trace sputum (PWTS) could aid clinical decision-making.

**Methods:**

We enrolled adults and adolescents with trace-positive sputum on initial TB diagnostic evaluation in Uganda and South Africa. Participants were extensively evaluated at enrollment; those with uncertain TB status were followed off treatment, with interval reevaluations by TB clinicians, for up to 3 months. We assessed TB prevalence and associated patient characteristics and diagnostic results.

**Results:**

Among 311 PWTS, TB was identified by sputum culture at enrollment in 20% of participants (61/311, 95% CI 15%–24%). Within 3 months, 48% (145/301, 95% CI 43%–54%) had been judged to warrant TB treatment, and among those followed until microbiologic outcomes, 30% (68/227, 95% CI 24%–36%) had positive culture and 41% (99/240, 95% CI 35%–47%) had positive culture or Ultra. Two participants died. Having TB symptoms, advanced human immunodeficiency virus (HIV), and no recent TB history were associated with microbiologically confirmed TB disease, as were an abnormal chest X-ray (in those without recent TB) or elevated C-reactive protein (CRP).

**Conclusions:**

Roughly half of PWTS were recommended TB therapy. This prevalence supports treating most PWTS when multimodal testing and repeated clinical evaluations are infeasible. However, given low observed mortality, some people with low-risk characteristics and negative results on other widely available diagnostic tests could safely defer treatment with clinical follow-up.

## Teaser

Half of patients with trace results on initial Xpert MTB/RIF Ultra diagnostic testing were diagnosed with tuberculosis (TB) within 3 months. Negative HIV status, recent TB treatment, negative chest X-ray, and low C-reactive protein identified patients at lower risk.

## Graphical Abstract

**Graphical Abstract:**
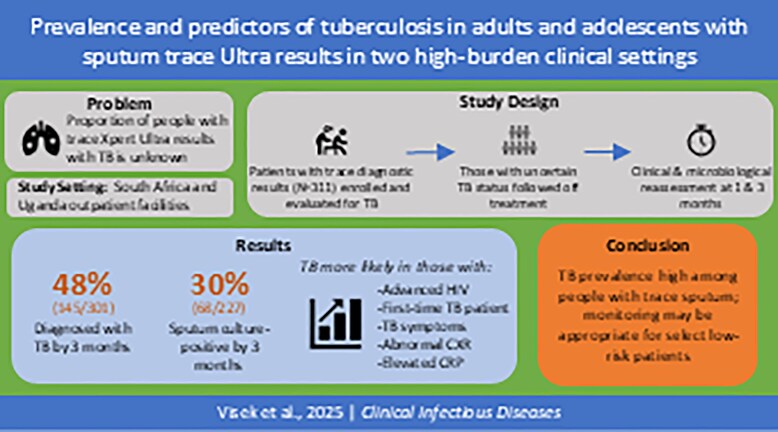

**(See the Editorial Commentary by Sossen and Martinson on pages e1311–2.)**

Tuberculosis (TB) detection remains a challenge globally, with an estimated 24% of cases never formally diagnosed [1]. Expanding access to rapid molecular diagnostics is a World Health Organization (WHO) priority, as they allow for accurate diagnosis of TB, early detection of drug resistance, and prompt initiation of effective therapy [1]. Xpert MTB/Rif Ultra (“Ultra”; Cepheid), a widely used rapid diagnostic test, achieves sensitivity approaching that of culture, partly through incorporation of multi-copy *Mycobacterium tuberculosis* (MTB) gene targets [2, 3]. Its lowest level of MTB detection, a category called “trace,” indicates detection of only these multi-copy targets and not the single-copy-per-genome *rpoB* target.

Trace results have uncertain clinical significance, as the presence of MTB DNA may reflect very low disease burden or remnant material from nonviable bacilli [4]. In diagnostic evaluations of patients with presumptive pulmonary TB, the trace category has accounted for 8%–41% of all positive Ultra results [5–8], and as many as 9 in 10 of these trace results were associated with negative sputum cultures and considered false positives [5]. However, most studies were small and included limited diagnostic evaluations. More extensive and repeated evaluations prior to treatment could clarify whether some trace results represent disease even when cultures are negative. Furthermore, apart from an association of previous TB treatment with false-positive results [6, 8, 9], little is known about how characteristics of people with trace sputum (PWTS) alter their likelihood of TB disease. Given these gaps in knowledge, WHO guidelines recommend treating all people with presumptive TB with trace results, with the narrow exception of human immunodeficiency virus (HIV)-negative adults treated for TB within the past 5 years [10]. Understanding which trace results are most likely to indicate TB disease would allow for more nuanced treatment decisions.

Therefore, we conducted a prospective cohort study of PWTS identified during routine TB diagnostic evaluations in 2 countries, to better delineate their TB disease status. If participants’ TB status remained uncertain after a systematic diagnostic evaluation, treatment was deferred, and they were reassessed 1 month and 3 months later. We estimated the proportions of people diagnosed with TB clinically or microbiologically within 3 months and identified patient characteristics and ancillary diagnostic test results associated with TB disease.

## METHODS

### Study Design, Participants, and Procedures

We recruited adult and adolescent PWTS from routine TB diagnostic evaluations at 7 health facilities in Kampala, Uganda, from February 2022 to July 2024, and from outpatient clinics of Harry Gwala Regional Hospital and its catchment area near Pietermaritzburg, South Africa, from November 2022 to June 2024. We attempted to enroll all eligible PWTS. Eligible patients had not yet initiated TB treatment, did not require hospitalization, and had not received TB treatment (both sites) or preventive therapy (South Africa only) within the previous 3 months. We targeted a sample size of ≥300 to provide precision of at least ±6% for estimating TB prevalence and >80% power for detecting meaningful risk–factor associations and differences between diagnostic algorithms (Supplementary Tables 1–3).

Within 14 days of the initial Ultra result, consenting participants underwent an extensive baseline evaluation that included a standardized interview, physical exam, digital chest X-ray (CXR), repeat sputum Ultra testing, 2 expectorated sputum cultures (separately sent for solid and liquid culture [South Africa] or each split for both liquid and solid culture [Uganda]), *Mtb* immunoreactivity testing (QuantiFERON-TB Gold+; Uganda only), chest computed tomography (CT) (Uganda only), HIV testing, and serum C-reactive protein (LumiraDx [South Africa], ichroma [Uganda]; CRP) measurement. People with HIV also underwent urine lipoarabinomannan (LAM) testing and CD4+ T-cell count measurement. Solid cultures were incubated for 8 weeks (both sites), and liquid cultures were held for 4–6 weeks (Uganda) or 6–8 weeks (South Africa).

Participants who remained untreated after this baseline diagnostic evaluation (by recommendation of a clinician or because the participant declined treatment) were reassessed 1 and 3 months later with interview, sputum Ultra, sputum culture, CRP, and (at 3 months) repeat CXR.

### Monitoring and Treatment Initiation

Local clinicians at each site made treatment decisions based on clinical judgment. In South Africa, a study physician evaluated each participant and determined whether to initiate or defer TB treatment; in ambiguous cases, decisions were made by consensus of a 3-physician panel. In Uganda, TB clinicians at referring health facilities could start treatment at any time but were encouraged to await study investigations when appropriate; 3-physician advisory panels also reviewed study data and made additional treatment recommendations. Investigators shared all study evaluations with physician panelists, and participants received written results to share with external clinicians.

Although follow-up continued for a year or more, this analysis focuses on TB diagnosed within 3 months, a period interpreted as indicating “prevalent” disease (present at the time of the initial trace result).

### Outcomes and Missing Data

Primary analyses use a composite TB reference standard: having MTB complex on sputum culture, a recommendation to start TB treatment (on any microbiological and/or clinical basis), or death attributed to TB. Sensitivity analyses use more limited microbiological (MTB complex on sputum culture or repeat Ultra positive > trace) and culture-only reference standards. All reference standards are assessed at 2 timepoints: at baseline (considering only results from, and treatment decisions based on, the baseline study visit) and within 3 months of enrollment (considering all participants diagnosed within 3 months to have had TB at the time of their initial trace result). Participants are excluded from any microbiological outcome for which results are missing or uninterpretable, from all 3-month outcomes if they missed the 3-month visit, and from microbiological outcomes at 3 months if they initiated treatment before the 3-month visit without microbiological confirmation (Supplementary Tables 4–6).

### Data Analysis

This was a prespecified analysis of prevalence using the 3-month time frame and TB definitions described above. Continuous variables are summarized as median and interquartile range (IQR). Prevalence estimates are reported with 95% Wald CIs.

We evaluated patient characteristics associated with TB using log-binomial and robust Poisson regression. We developed analogous models for each of the 3 TB reference standards, classifying by the culture-only reference at baseline (to avoid bias from earlier culture-negative TB diagnoses) and the composite and microbiological reference standards at 3 months. We used forward stepwise selection of patient characteristic variables for each reference standard separately, considering the following prespecified variables for inclusion: age (dichotomized at 35 years), gender, country, history of tobacco smoking, recent TB (treated within the past 5 years, as categorized by WHO [10]), symptoms (cough, weight loss, night sweats, or fever at baseline), recent household TB contact (diagnosed within the past 6 months), HIV status, and CD4 count (modeled only as an interaction with HIV status and categorized using the commonly accepted threshold for advanced HIV [11] as <200 cells/mm^3^, ≥200 cells/mm^3^, or unknown). Variables selected for any reference standard were included in all final models. A small number of participants with missing covariate data (Table 1) were excluded, except in a sensitivity analysis using multiple imputation (“mice” package; Supplementary Methods).

**Table 1.** Patient Characteristics and Baseline Diagnostic Results for Trace-Positive Participants

Variable	Combined Cohort (N = 311)	Uganda (N = 170, 55%)	South Africa (N = 141, 45%)
Age, years (median [IQR])	37 [30, 47]	35 [29, 43]	40 [33, 53]
Male sex (N [%])	155 (50)	98 (58)	57 (40)
Symptoms present on initial screen			
Cough (N [%])	243 (78)	154 (91)	89 (63)
Fever (N [%])	118 (38)	71 (42)	47 (37)
Night sweats (N [%])	88 (28)	42 (25)	46 (33)
Weight loss (N [%])	146 (47)	99 (58)	47 (33)
Underweight (BMI <18.5) (N [%])	52 (17%)	37 (22%)	15 (11%)
HIV positive (N [%])	178 (57)	75 (44)	103 (73)
CD4 count < 200 cells/mm^3^ (N [%])	35 (21)	16 (24)	19 (19)
CD4 count > 200 cells/mm^3^ (N [%])	133 (79)	50 (76)	83 (82)
Taking ART (N [%])	144 (86)	57 (78)	87 (92)
Pregnant (N [%])	11 (4)	4 (2)	7 (5)
Smoking status			
Never (N [%])	203 (65)	116 (68)	87 (62)
Current (N [%])	59 (19)	18 (11)	41 (29)
Former (N [%])	49 (16)	36 (21)	13 (9)
Smoking history of 20 or more pack-years among current or former smokers (N [%])	69 (73)	37 (69)	32 (78)
Alcohol use: Positive AUDIT-C score^a^ (N [%])	104 (33)	55 (32)	49 (35)
Prior TB treatment (N [%])	113 (36)	64 (38)	49 (35)
Prior TB treated within the last 5 y (N [%])	72 (24)	49 (29)	23 (17)
Recent household contact with TB (within the past 6 m) (N [%])	25 (8)	13 (8)	12 (9)
Abnormal baseline chest X-ray result (N [%])	196 (65)	111 (68)	85 (61)
Baseline laboratory evaluation			
C-reactive protein >5 mg/L (N [%])	155 (50)	85 (50)	70 (50)
Repeat Xpert Ultra result			
Negative (N [%])	215 (71)	117 (69)	98 (74)
Trace (N [%])	35 (12)	22 (13)	13 (10)
Positive (>trace) (N [%])	52 (17)	31 (18)	21 (16)
Positive culture (N [%])	61 (20)	41 (24)	20 (14)
Positive urine lipoarabinomannan (N [%])^b^	11 (7)	4 (10)	7 (7)

Small number of participants were missing data for: underweight (3), CD4 count among participants with HIV (10), ART status among participants with HIV (10), pregnancy status among female participants (1), pack-year history among current or former smokers (13), history of prior TB (1), timing of prior TB treatment among participants with a history of TB (6), baseline CXR (9), C-reactive protein (1), repeat Xpert Ultra (9), and urine lipoarabinomannan among participants with HIV (4). Missing data was excluded from the denominators when calculating percentages.

Abbreviations: ART, antiretroviral therapy; AUDIT-C, Alcohol Use Disorders Identification Test; BMI, body mass index; HIV, human immunodeficiency virus; IQR, interquartile range; TB, tuberculosis.

^a^Positive AUDIT-C score defined as 4 or greater for men and 3 or greater for women.

^b^Reported for participants with HIV only.

To the resulting “patient characteristics” models, we subsequently added either CXR results (normal/abnormal, “CXR-added model”) or CRP results (normal/elevated using a threshold of 5 mg/L, “CRP-added model”) as an additional covariate. The CXR-added model included an interaction between CXR result and recent TB. We generated receiver operating characteristic (ROC) curves for each model and calculated areas under the ROC curves (AUCs) using the “pROC” package (version 1.18.5).

Finally, we used our CXR-added regression model to estimate the risk of TB among a simulated cohort of patients with a specified set of low-risk features—namely, negative HIV status and normal baseline CXR—and with no history of TB. Results are reported as the median and IQR of estimated individual-level risks. See Supplementary Methods for further detail.

All analyses used R Statistical Software version 4.3.1 (R Core Team, 2023).

### Ethical Approval Statement

Ethical review boards at the University of KwaZulu-Natal, the Makerere University School of Public Health, University of Washington, and Johns Hopkins University approved the study.

## RESULTS

A total of 311 PWTS (170 in Uganda; 141 in South Africa) were enrolled by the analysis cutoff dates. Another 796 (59 in Uganda; 737 in South Africa) had trace results but were not enrolled, primarily due to distance or inability to make contact (Figure 1, Supplementary Figure 1). At 1 month, 202 participants remained alive and untreated, and 190 completed the follow-up visit; at 3 months, these figures were 170 and 162, respectively. Mortality during this 3-month period was 0.6% (2/311), with 1 death attributed to TB.

**Figure 1.**
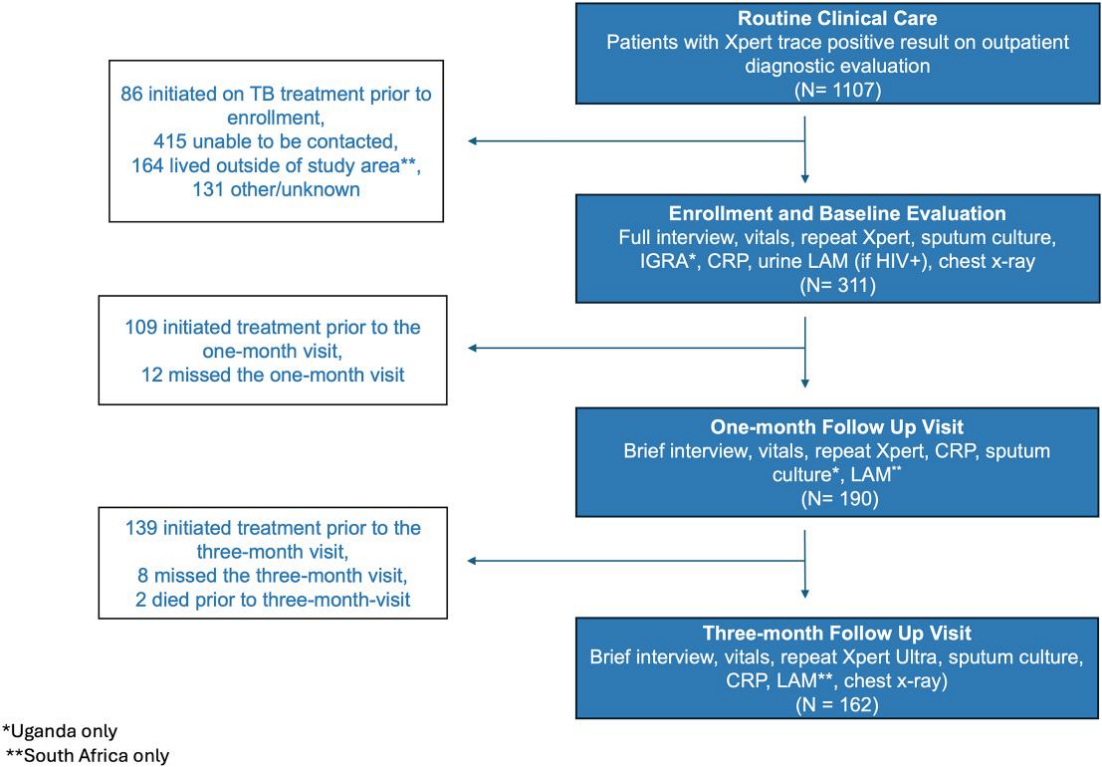
Study population overview. Flow diagram of patients with trace results on diagnostic evaluation, study enrollment, and visit completion, with reasons for exclusion. Abbreviations: CRP, c-reactive protein; HIV, human immunodeficiency virus; LAM, lipoarabinomannan; TB, tuberculosis. The meaning of the abbreviation ‘ IGRA’ to be added.

The cohort was 50% male (155/311) with a median age of 37 (Table 1). HIV prevalence was 57% (178/311), and most participants with HIV were on antiretroviral therapy (ART) and had CD4 counts of ≥200 cells/mm^3^. Nearly a quarter (24%) had been treated for TB within the past 5 years, and most had 1 or more TB symptoms. On repeat Ultra testing at baseline, most participants (215/302; 71%) tested negative, but 12% (35/302) had a trace result (of whom 11 [31%] also had positive baseline sputum cultures) and 17% (52/302) had positive results > trace (of whom 32 [62%] had positive cultures). Half of participants (50%; 156/310) had an elevated CRP, and 65% (196/302) had an abnormal CXR (Table 1).

Overall, 145 of 301 participants (48%, 95% CI 43%–54%) were positive by the composite reference standard by 3 months (Figure 2). While the majority were diagnosed through the baseline evaluation, 25 (17%) of these diagnoses were based on additional microbiological, clinical, and/or radiographic data obtained during follow-up. Eighty-three (57%) were confirmed by Ultra (positive > trace) or culture (Supplementary Figure 2).

**Figure 2.**
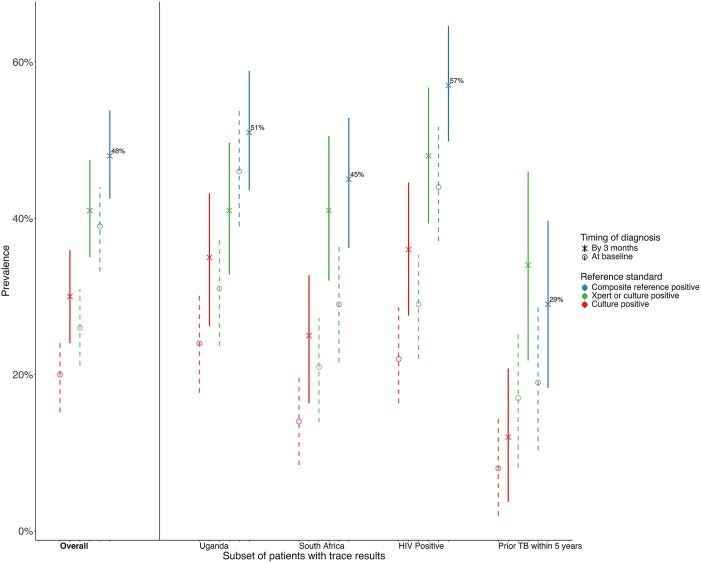
TB prevalence among patients with initial trace Xpert Ultra results, by multiple definitions. TB prevalence by the composite, microbiological (Xpert or culture), and sputum culture-only reference standards assessed at baseline and by 3 months, for the overall cohort and several subgroups. Prevalence estimates are represented as a point estimate and plotted with lines showing their 95% CIs. CD4 count-stratified and symptom-stratified results are shown in Supplementary Figure 3. Abbreviations: CI, confidence interval; HIV, human immunodeficiency virus; TB, tuberculosis.

Of those who could be evaluated using the microbiological reference standard, TB prevalence was 26% (81/311, 95% CI 21–31%) at baseline and 41% (99/240, 95% CI 35–47%) by 3 months (Figure 2). Some patients with positive microbiological classifications (31/99 [31%] of those positive by 3 months) had positive (>trace) Ultra results but negative cultures; 16 of these were considered falsely positive with no treatment recommended. When considering sputum culture only, TB prevalence was 20% (61/311, 95% CI 15%–24%) at baseline. Among the entire cohort, 68/311 (22%) had at least 1 positive culture by 3 months, with 7 participants having newly positive cultures during follow-up; 3-month prevalence by the culture reference standard (after excluding 84 who had started treatment without culture confirmation [72 participants] or were missing 3-month culture results [12]) was 30% (68/227, 95% CI 24%–36%).

In univariate analysis, TB was associated with male gender, HIV positivity, low or unknown CD4 count, TB symptoms, and a lack of recent TB treatment history, as well as with diagnostic results of abnormal CXR (particularly in those without recent TB) and elevated CRP (Table 2; Supplementary Tables 9–10). A positive or trace repeat Ultra was associated with a TB prevalence ratio (PR) of 2.2 (95% CI 1.8–2.7) for the 3-month composite reference standard and 5.9 (95% CI 3.6–9.6) by the baseline culture-positive reference standard (Supplementary Table 7).

**Table 2.** Multivariate Log-Binomial Regression Model Coefficients for Discriminating TB Versus No TB, Using Composite Reference Standard Over 3 Months

Covariate	Unadjusted Prevalence Ratio (95% CI), n = 287^c^	Adjusted Prevalence Ratio (95% CI) for Variables Included in Final Model		
		Patient characteristics model, n = 295^d^	CXR-added model, n = 288^e^	CRP-added model, n = 294^f^
Uganda site	1.2 (1.0, 1.6)	…	…	…
Age < 35	1.1 (0.9, 1.5)	1.2 (0.8, 1.6)	1.2 (0.8, 1.7)	1.2 (0.8, 1.6)
Male gender	1.3 (1.0, 1.7)	1.3 (0.9, 1.8)	1.2 (0.8, 1.7)	1.2 (0.9, 1.7)
History of prior TB within 5 y	0.5 (0.3, 0.8)	0.6 (0.3, 0.9)	0.9 (0.1, 2.9)	0.6 (0.4, 0.9)
Presence of 1 or more TB symptom^a^	3.9 (1.9, 10.4)	3.1 (1.5, 7.8)	3 (1.3, 8.5)	2.7 (1.3, 7.1)
Recent household TB contact^b^	0.7 (0.4, 1.2)	0.8 (0.4, 1.5)	0.9 (0.4, 1.7)	0.8 (0.4, 1.5)
Any smoking history	1.1 (0.9, 1.4)	…	…	…
HIV positive (all)	1.6 (1.2, 2.1)	…	…	…
HIV positive with CD4 < 200 cells/mm^3^	2.5 (1.5, 4.0)	2.0 (1.2, 3.3)	1.9 (1.2, 3.2)	1.7 (1.1, 2.9)
HIV positive with CD4 > 200 cells/mm^3^	1.3 (0.9, 2.0)	1.4 (0.9, 2.1)	1.4 (1, 2.2)	1.5 (1.0, 2.2)
HIV positive with CD4 unknown	2.5 (1.1, 5.1)	2.0 (0.9, 4.1)	2.3 (1.0, 4.7)	2.0 (0.9, 4.2)
Abnormal CXR (overall)	1.7 (1.3, 2.4)	*…*	…	*…*
Abnormal CXR in those without history of TB within 5 y	2.1 (1.4, 3.2)	…	1.8 (1.2, 2.8)	…
Abnormal CXR in those with history of TB within 5 y	0.4 (0.1, 2.6)	…	0.6 (0.1, 3.7)	…
CRP > 5 mg/L	2.0 (1.6, 2.7)	*…*	…	1.7 (1.2, 2.5)

Abbreviations: CXR, chest X-ray; HIV, human immunodeficiency virus; TB, tuberculosis.

^a^Defined as cough, fever, weight loss, or night sweats at the time of study enrollment.

^b^Defined as living with someone diagnosed with TB within the past 6 m.

^c^24 observations dropped due to incomplete data.

^d^16 observations dropped due to incomplete data.

^e^23 observations dropped due to incomplete data.

^f^17 observations dropped due to incomplete data.

n multivariable regression, TB (defined by the composite reference standard by 3 months) remained positively associated with advanced HIV (CD4 < 200 cells/mm^3^: PR 2.0 [95% CI 1.2–3.3]) and TB symptoms (PR 3.1 [95% CI 1.5–7.8]) and inversely associated with recent TB (PR 0.6 [95% CI .3–.9]) (Table 2). When considering additional diagnostic test results, either an abnormal CXR (in those without recent TB: PR 1.8 [95% CI 1.2–2.8]) or an elevated CRP (PR 1.7 [95% CI 1.2–2.5]) was associated with greater likelihood of TB. Results for alternative reference standards were largely similar, though associations with TB history and CXR results were weaker when using the microbiological reference standard (Supplementary Tables 9–10). A sensitivity analysis using multiple imputation produced generally similar results (Supplementary Table 11).

Patient characteristics classified TB status (composite reference standard, 3 months) with an AUC of 0.76 (95% CI .71–.82). This AUC increased to 0.80 (95% CI .75–.85) or 0.79 (95% CI .74–.84) after adding CXR or CRP, respectively (Figure 3). Areas under the ROC curves were similar for the other reference standards (Supplementary Figure 4).

**Figure 3.**
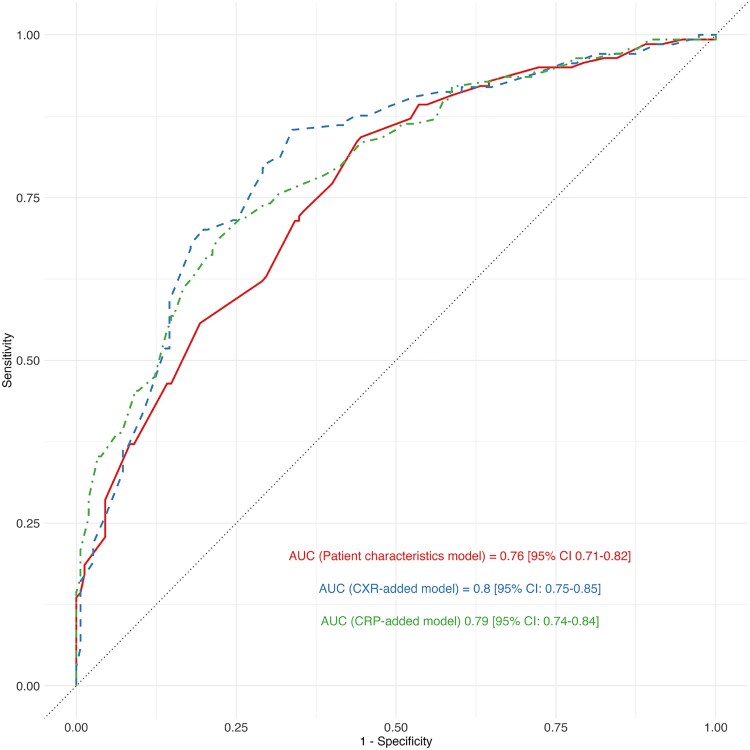
Receiver operating characteristic curves for the basic characteristics, CXR-added, and CRP-added models using the composite reference standard over 3 m. Abbreviations: AUC, area under the curve; CI, confidence interval; CRP, c-reactive protein; CXR, chest X-ray.

When we applied the CXR-added regression model to the simulated “low-risk” cohort of HIV-negative patients with normal baseline CXRs (and no recent history of TB), the median risk of TB (by the composite reference standard) was 27% (IQR, 22%–30%).

## DISCUSSION

Our study examines the largest cohort to date of patients with trace Ultra sputum diagnostic test results. More than 300 patients from multiple facilities in 2 high-prevalence countries were followed untreated for up to 3 months to clarify their TB status. Nearly half were diagnosed with TB within 3 months, including 41% with microbiological confirmation. Baseline sputum culture (on which 20% of patients tested positive) did not capture the full burden of TB. Still, half of patients did not have sufficient evidence to diagnose TB despite 3 months of extensive testing. Across multiple TB definitions, patient characteristics associated with TB in this population included advanced HIV, TB symptoms at the time of enrollment, and having no recent history of TB treatment. Chest X-ray and CRP testing results provided further risk stratification.

Defining TB disease is challenging. Sputum culture can be insufficiently sensitive to detect active pulmonary disease with low bacillary burden [12]. Tuberculosis disease burden may also wax and wane [13, 14], limiting detection through 1-time evaluations. We considered TB diagnosed within 3 months of a trace result, a timeframe chosen to allow for multiple reassessment opportunities and sufficient disease progression in cases of recent primary exposure, yet short enough that any TB that was identified was likely to explain the initial trace result. Using multiple TB reference standards and repeated evaluations allowed us to more fully capture active disease near the limit of detection and demonstrated consistent associations across TB definitions.

The TB burden estimated in our trace cohort is similar or higher compared to others, which is compared against culture [5–9]. While TB prevalence varies by definition, our study's most conservative estimate is 22%, the proportion of our originally enrolled cohort (68/311) with a positive culture within 3 months. Our composite (48%) and microbiological (41%) prevalence estimates provide upper bounds but may be inflated by clinical overdiagnosis and false-positive Ultra results, respectively. Our results may underestimate TB prevalence among all PWTS, because 2 excluded groups—those empirically treated for TB before test results returned and those requiring hospitalization—may have particularly high likelihood of having TB disease. Our prevalence estimates are high enough to support treatment for most PWTS in high-burden settings when further testing and follow-up is not feasible.

We identified characteristics significantly associated with TB among PWTS. Consistent with other studies [6, 8, 9], we found that trace results in patients with a recent history of TB treatment were less likely to indicate current active disease. Although most participants reported symptoms, being asymptomatic at baseline conferred a roughly 3-fold lower risk. People with trace sputum identified through targeted universal TB testing strategies [15, 16] may therefore be less likely to have TB than those identified through symptom-driven testing. Finally, we found that advanced HIV was associated with microbiologically and culture-confirmed TB among PWTS. This finding supports current WHO guidelines’ recommendation to treat people with HIV with a trace result [10], not only due to elevated risk for serious illness if TB goes untreated but also due to a higher TB burden.

Our results also suggest that additional diagnostic testing after a trace result can inform TB risk. A normal CXR (in those without recent TB) and a normal CRP were associated with a 1.5–3-fold lower TB risk across multiple definitions, although the absolute improvement in TB status classifications (as measured by AUC) was small. In addition, our results fill a previously identified evidence gap [17] by indicating that a negative result on a repeat Ultra test reduces TB likelihood by at least half, depending on the reference standard (Supplementary Table 7). Therefore, although we cannot estimate predictive accuracy within all PWTS subgroups, our cohort-wide results suggest that a negative diagnostic result on any of several widely available diagnostic tests—CXR, CRP, and repeat Ultra—could support deferring treatment for an otherwise low-risk patient near treatment thresholds (Supplementary Table 8). Reassuringly, external evidence also suggests that deferring treating for patients with low quantitative Ultra results contributes little to transmission [18].

Our study has several limitations. First, we lack clinical and demographic data to assess how nonenrolled PWTS differed from our enrolled cohort. Although much of the nonenrollment was due to distance or lack of contact information, limiting enrollment to patients who had not yet initiated TB treatment may have disproportionately excluded more ill patients (especially in South Africa, where nearly 1 in 10 PWTS started treatment before they could be recruited). Therefore, our results apply primarily to nonhospitalized patients in high-incidence settings whose TB status is sufficiently uncertain that a treating clinician would defer empiric treatment pending test results. Similarly, among those enrolled, participants who were more ill were more likely to be clinically diagnosed early in follow-up, limiting later opportunities to detect MTB microbiologically and potentially biasing the 3-month microbiological-based prevalence estimates. In addition, differences may have existed by study site in microbiological testing sensitivity (eg the second sputum culture specimen was collected in the early morning in Uganda, where baseline culture-positive prevalence was higher) and in clinicians’ threshold for clinical diagnosis (particularly in the context of HIV coinfection). Notably, national guidelines in Uganda advise treatment for PWTS, while South African guidelines defer to clinician judgment [16, 19]. In both settings, clinicians made treatment decisions with knowledge of the study's intensive follow-up protocol and likely deferred treatment for patients whom they would have treated under usual circumstances. Finally, the 3-month timeframe could be too brief to detect slowly progressing disease.

In conclusion, our study suggests that the TB prevalence among PWTS is high, but that up to half do not have clinically or microbiologically evident TB even with 3 months of repeated culture, molecular testing, and clinical evaluations. A prevalence of half supports the current practice of treating most PWTS, but treatment deferral may be appropriate in select patients with lower-risk characteristics or reassuring results on additional diagnostic testing such as X-ray, inflammatory markers, or further molecular testing, particularly when close clinical follow-up is feasible.

## Supplementary Material

ciag019_Supplementary_Data

## Supplementary Data

Supplementary materials are available at *Clinical Infectious Diseases* online. Consisting of data provided by the authors to benefit the reader, the posted materials are not copyedited and are the sole responsibility of the authors, so questions or comments should be addressed to the corresponding author.
